# Utility Of An Emergency Department Clinical Protocol For Early Identification of Coronavirus Infection

**DOI:** 10.5811/westjem.2020.12.49470

**Published:** 2021-04-05

**Authors:** William Bonadio, Kaedrea Jackson, Lindsey Gottlieb, Eric Legome

**Affiliations:** Mount Sinai Medical Center, Department of Emergency Medicine, New York City, New York

## Abstract

**Introduction:**

We assessed the utility of an emergency department (ED) protocol using clinical parameters to rapidly distinguish likelihood of novel coronavirus 2019 (COVID-19) infection; the applicability aimed to stratify infectious-risk pre-polymerase chain reaction (PCR) test results and accurately guide early patient cohorting decisions.

**Methods:**

We performed this prospective study over a two-month period during the initial surge of the 2020 COVID-19 pandemic in a busy urban ED of patients presenting with respiratory symptoms who were admitted for in-patient care. Per protocol, each patient received assessment consisting of five clinical parameters: presence of fever; hypoxia; cough; shortness of breath/dyspnea; and performance of a chest radiograph to assess for bilateral pulmonary infiltrates. All patients received nasopharyngeal COVID-19 PCR testing.

**Results:**

Of 283 patients studied, 221 (78%) were PCR+ and 62 (22%) PCR-. Chest radiograph revealed bilateral pulmonary infiltrates in 85%, which was significantly more common in PCR+ (94%) vs PCR- (52%) patients (P < 0.0001). The rate of manifesting all five positive clinical parameters was significantly greater in PCR+ (63%) vs PCR- (6.5%) patients (P < 0.0001). For PCR+ outcome, the presence of all five positive clinical parameters had a specificity of 94%, positive predictive value of 98%, and positive likelihood ratio of 10.

**Conclusions:**

Using an ED protocol to rapidly assess five clinical parameters accurately distinguishes likelihood of COVID-19 infection prior to PCR test results, and can be used to augment early patient cohorting decisions.

## INTRODUCTION

During the 2020 coronavirus 2019 (COVID-19) pandemic, urban hospitals experienced excessively high patient volumes and significant spatial constraints.^1^ Emergency departments (ED) struggled to manage the acute patient influx, particularly given the continued circulation of influenza and other respiratory viruses early in the pandemic. Basic epidemiologic care principles support cohorting patients with like infectious status to reduce risk of nosocomial transmission.^2^,^3^ Specifically, it is important to avoid cohorting a COVID-19 person under investigation (PUI) who is not infected with confirmed COVID-19 cases. Routine processing polymerase chain reaction (PCR) tests can take up to 24 hours; thus, waiting for test results to make cohorting decisions poses an unacceptable burden on the capacity to care for PUIs. While more rapid testing platforms are being developed, it is not clear when, and how widely, they will be made available; or how accurate they will be.

In the meantime, implementing a clinical protocol that accurately allows cohorting presumptively positive PUIs with known COVID-19 positive patients while awaiting inpatient bed assignment would optimize utilization of nursing, physical space, and physician oversight. Such a protocol would also ensure that PUIs with lower pre-PCR test probability of COVID-19 infection remain isolated apart from confirmed COVID-19 cases, thus decreasing the risk of nosocomial transmission.

We developed a simple COVID-19 ED screening protocol consisting of five discriminatory, commonly assessed clinical parameters (including performance of a chest radiograph [CXR]). The objective of this study was to prospectively evaluate this screening protocol to predict the likelihood of PCR+ for COVID-19 infection prior to PCR test resulting. We hypothesized that during times of high COVID-19 community prevalence, this clinical protocol would facilitate early and accurate identification of PUIs at risk for COVID-19 infection, allowing them to cohort with known infected patients while awaiting results of the PCR test.

## METHODS

During the initial surge of the 2020 COVID-19 pandemic, from March 1–April 28, we performed a prospective study of patients presenting to our urban ED, which treats >100,000 patients per year. In late February 2020, in conjunction with our infection control department, an ED protocol was devised and implemented anticipating that spatial constraints would eventually amaze the ability to provide appropriate isolation distancing between PUIs and known COVID-19 infected patients. This protocol was applied as standard practice during the study period to inform active clinical decision-making regarding cohorting of admitted/boarded ED patients.

All patients aged 30–70 years presenting with acute respiratory symptoms consistent with possible COVID-19 infection as judged by an attending-level emergency physician were screened by initial providers using parameters given in Table 1. Since the protocol was devised prior to the release of the US Centers for Disease Control and Prevention (CDC) demographic risk information, we arbitrarily set an upper age limit at 70 years. Our electronic health record (EHR) (Epic Systems Corporation, Verona, WI) used an extensive standardized template, including querying whether patients experienced fever, shortness of breath, and cough. Standard triage protocol mandated performance of pulse oximeter 0_2_ saturation measurement and measurement of body temperature in all patients. The EHR was reviewed in its entirety, noting all entries made by all providers.

**Population Health Research Capsule**

What do we already know about this issue?

*There is little published literature defining an emergency department (ED) clinical scoring system to define risk for COVID-19 infection in patients who present with respiratory symptoms during a pandemic.*

What was the research question?

*Can a clinical protocol accurately identify ED patients with COVID-19 infection to facilitate cohorting with known infected patients while awaiting polymerase chain reaction (PCR) test results?*

What was the major finding of the study?

*An ED protocol assessing 5 clinical parameters accurately distinguishes COVID-19 infection risk prior to PCR test results to augment early patient cohorting decisions.*

How does this improve population health?

*Utilizing this clinical protocol facilitates early and accurate identification of risk for COVID-19 infection.*

We used a clinical decision tool composed of five variables: 1) hypoxia (O_2_ saturation ≤92% on room air while in the ED or required supplemental oxygen to maintain adequate O_2_ saturation); 2) fever, either by history (≥100.4° Fahrenheit) or measured in the ED (≥38° Celsius); 3) cough; 4) dyspnea/shortness of breath (SOB); and 5) CXR with bilateral pulmonary infiltrates. Every effort was made by study investigators to follow the EHR census in real time to screen/enroll consecutively presenting patients appropriate for study. In addition, the EHR was reviewed every 24 hours to compile a list of consecutive admissions. Initial clinical parameters were tabulated up to 24 hours prior to PCR test results, and included symptomatology (presence of fever, cough, dyspnea/SOB), and vital signs measurements (body temperature and pulse oximeter O_2_ %-saturation). All received a COVID-19 nasopharyngeal qualitative PCR test (“SARS-CoV-2 PCR” (Roche Laboratories Inc, Rotkreuz, Switzerland) and expedited ED CXR. PCR test results were reviewed and recorded when completed on the next calendar day after presentation.

Consistent with the intended use of the guidelines, we initially surveyed a sample of all patients admitted/boarded in the ED who were awaiting PCR test results, whose medical records were reviewed to determine protocol utility and efficacy. This was performed as a quality assurance project. Two authors (EL and WB) entered patients into the study independently; WB entered the majority, and EL reviewed all entries prior to finalizing data. There were only two discrepancies, both of which were removed from the final analysis.

### Statistical Analysis

We performed chi-squared or Fisher’s exact test to assess the significance of rate differences characterizing the presence of all five positive clinical variables between COVID19 outcome groups, using *P* ≤ 0.05 as the significance level (MEDCALC Software Ltd, Ostend, Belgium). We calculated sensitivity, specificity, positive/negative predictive values, and likelihood ratios.^4^,^5^

### Power Analysis

A sample of 76 cases was calculated to allow for 80% power (alpha 0.05) to determine the significance of difference in rates of all five positive clinical variables being present between PCR+ (estimated 50%) vs PCR- (estimated 20%) groups. The study was approved by our institutional review board.

## RESULTS

There were 283 consecutive admitted ED patients studied during the two-month period, of whom 221 (78%) were PCR+ and 62 (22%) PCR-. The duration of symptoms ranged between 1–28 days. All patient records had a provider entry for history of fever, SOB, and cough as queried by nursing at triage, and also by an attending-level emergency physician at the point of initial examination. Also, in each case there was standardized documentation of vital signs including triage measurements of body temperature and pulse oximeter 0_2_ saturation, and a CXR was performed early in the course of ED care, with results interpreted by an attending radiologist.

Table 2 gives patient clinical characteristics. Table 3 shows the distribution of clinical parameters per PCR result; overall, the rate of manifesting all five clinical parameters was significantly greater in PCR+ (63%) vs PCR- (6.5%) patients (*P* < 0.0001). The rate of radiographically identified bilateral pulmonary infiltrates was significantly greater in PCR+ (94%) vs PCR- (52%) patients (*P* < 0.0001). Table 4 gives results of statistical analysis; the manifestation of all five clinical parameters was highly predictive of PCR+ outcome, with a positive likelihood ratio of 10.

## DISCUSSION

The COVID-19 global pandemic presented many unique ED-resource challenges in managing a critical patient census, often requiring precautionary PUI isolation pending PCR test confirmation. As was the situation for many hospitals providing care, PUIs who are pending COVID-19 PCR results may reside in the ED for hours. Such was the case in our ED; at peak prevalence, we simultaneously boarded >60 COVID-19 admitted patients. Bed space was certainly at a premium, and the issue of accurate PUI cohorting based on infectious status was of primary importance. While awaiting PCR test results, providers had to subjectively determine (with variable accuracy) optimal patient placement based on an estimated likelihood of COVID-19 infection.

There are many advantages to early and accurate determination of patient COVID-19 infectious status, including preventing nosocomial infection, maximizing efficient utilization of limited bed space and PPE equipment, and augmenting contact tracing efforts. We were unable to identify prior published data analyzing utility of an ED protocol using clinical parameters to accurately distinguish COVID-19 PUI infection risk. Nor were there any standard published guidelines endorsing ED screening criteria to determine patient cohorting during a critical census surge when PUIs are admitted/boarded. Recently published studies^6^–^12^ retrospectively reported rates of individual clinical variables for patients with COVID-19 infection. One^6^ produced a prediction model to help define overall risk for COVID-19 infection, although it used blood test results, which can take a variable amount of time to process.

The World Health Organization (WHO) endorses immediate isolation of PUIs for COVID-19 infection. Its diagnostic criteria^13^ includes the presence of an acute respiratory infection with at least one of the following symptoms: cough; sore throat; SOB; coryza; or anosmia; with or without fever. We refined this list to enhance timely assessment in accurately cohorting PUIs pre-PCR results, selecting common COVID-19 clinical variables endorsed by WHO and extending its criteria to include parameters of fever, hypoxia, and bilateral pulmonary infiltrates.

Our roster consisted of simple, standard variables routinely assessed by initial providers with each patient encounter, plus performance of pulse oximetry and CXR. The protocol cutoff point chosen to distinguish risk was highly applicable, as just over 50% of all presenting patients manifested all five clinical parameters. It accurately predicted risk for COVID-19 PCR+, as the presence of all five positive clinical parameters was associated with very high specificity, positive predictive value, and a10-fold positive likelihood ratio for COVID-19 infection.

## LIMITATIONS

Our protocol accurately determined risk for positive COVID-19 PCR test result. We did not seek to identify low-risk criteria for identifying those who are PCR-negative. A recently published study analyzed a useful scoring system and devised a calculator to determine overall risk for COVID-19 infection and may have utility to this end.^14^ Those who manifest all five clinical criteria (yet are PCR-negative) although rarely occurring, present a diagnostic dilemma. These patients may still be clinically suspected of COVID-19 infection, prompting either repeat PCR testing, performance of a full battery of COVID-19 blood tests (C-reactive protein, D-dimer, ferritin, troponin, etc.) to help further confirm COVID-19 status. Finally, we limited our analysis to those aged 30-70 years old, as we lacked demographic information determining likely age groups to contract COVID-19 infection at the time we devised and implemented the protocol. Although we anticipated our protocol would accurately apply to an older aged demographic, further study is warranted to assess this.

## CONCLUSION

We conclude that an ED screening protocol consisting of five basic clinical parameters is simple to use, rapidly completed, and accurate in distinguishing persons under investigation risk for COVID-19 infection prior to PCR test results. We recommend its use to augment cohorting accuracy when PUIs for COVID-19 are ED admitted/boarded during a critical census surge.

## Figures and Tables

**Table 1:** Emergency department protocol: five clinical parameters used to determine likelihood of COVID-19 positive polymerase chain reaction rest.

Cough Dyspnea/shortness of breath Fever Hypoxia Chest radiograph with bilateral pulmonary infiltrates

*COVID-19*, coronavirus disease 2019.

**Table 2:** Clinical characteristics of 283 suspected cases of COVID-19 admitted to the hospital.

Variables	N (%)
PCR +	221 (78%)
PCR−	62 (22%)
Bilateral pulmonary infiltrates	240 (85%)
Manifested all 5 positive clinical parameters (fever, cough, dyspnea/SOB, hypoxia, bilateral pulmonary infiltrates)	143 (51%)

*COVID-19*, coronavirus disease 2019; *PCR+*, positive polymerase chain reaction test; *PCR-*, negative polymerase chain reaction test; *SOB*, shortness of breath.

**Table 3:** Distribution of protocol clinical parameters based on COVID-19 PCR test result.

	PCR+ (N = 221)	PCR− (N = 62)
Bilateral pulmonary infiltrates	208 (94%)	32 (52%)
Hypoxia	185 (84%)	37 (60%)
Fever	169 (77%)	23 (37%)
Cough	206 (93%)	44 (71%)
Dyspnea/SOB	209 (95%)	59 (95%)
Manifested all 5 positive clinical parameters (fever, cough dyspnea/SOB, hypoxia, bilateral pulmonary infiltrates)	139 (63%)	4 (6.5%)

*COVID-19*, coronavirus disease 2019; *PCR+*, positive polymerase chain reaction test; *PCR*−, negative polymerase chain reaction test; *SOB*, shortness of breath.

**Table 4:** Statistical analysis for predicting COVID-19 PCR+ outcome with manifesting all five positive clinical parameters.

Statistic	Value	95% CI
Sensitivity	63%	(56 – 69%)
Specificity	94%	(84 – 98%)
Positive likelihood ratio	10	(3.7 – 25)
Negative likelihood ratio	0.4	(0.33 – 0.48)
Positive predictive value	98%	(94 – 99%)
Negative predictive value	39%	(34 – 43%)

*COVID-19*, coronavirus disease 2019, *PCR+*, positive polymerase chain reaction test; *CI*, confidence interval.
